# Exercise Modalities to Preserve Muscle Mass and Bone Health After Metabolic Bariatric Surgery

**DOI:** 10.1002/jcsm.70289

**Published:** 2026-04-30

**Authors:** Ariela Goldenshluger, Lior Friedman, Tamir Turjeman, Roi Yavetz, Asnat Raziel, David Goitein, Irit Markus, Hassan Kais, Ron Sternfeld, Estherina Trachtenberg, Gal Dubnov‐Raz, Ilanit Mhaler, Ilan Youngster, David Peled, Roee Amedi, Andrei Keidar, Edward H. Livingston, Nasser Sakran, Dror Dicker, Yftach Gepner

**Affiliations:** ^1^ Department of Epidemiology and Preventive Medicine, School of Public Health, Faculty of Medicine and Health Science and Sylvan Adams Sports Institute Tel Aviv University Tel Aviv Israel; ^2^ Endocrinology & Metabolism Institute Cleveland Clinic Cleveland Ohio USA; ^3^ Assuta Medical Center Tel Aviv Israel; ^4^ Faculty of Medicine and Health Sciences Tel Aviv University Tel Aviv Israel; ^5^ Department of General Surgery Sheba Medical Center Ramat Gan Israel; ^6^ Herzliya Medical Center Herzliya Israel; ^7^ Sagol School of Neuroscience Tel Aviv University Tel Aviv Israel; ^8^ Pediatric Exercise and Lifestyle Clinic, Safra Children's Hospital Tel HaShomer Sheba Medical Center Ramat Gan Israel; ^9^ Pediatric Infectious Diseases Unit and the Center for Microbiome Research Shamir Medical Center Be'er Ya'akov Israel; ^10^ Division of General Surgery Tel Aviv Sourasky Medical Center Tel Aviv Israel; ^11^ Department of Surgery, Faculty of Health Sciences UCLA School of Medicine Los Angeles California USA; ^12^ Department of General Surgery Holy Family Hospital Nazareth Israel; ^13^ The Azrieli Faculty of Medicine Bar‐Ilan University Ramat Gan Israel; ^14^ Internal Medicine and Obesity Clinic Hasharon Hospital‐Rabin Medical Center Petah Tikva Israel

**Keywords:** body composition, bone mineral density, exercise, fat‐free mass, metabolic bariatric surgery, physical function

## Abstract

**Background:**

Metabolic bariatric surgery (MBS) effectively reduces fat mass (FM), but how to best preserve muscle and bone mass after MBS is unknown.

**Objective:**

This study aimed to evaluate the effect of aerobic, resistance and combined exercise regimens on body composition, bone mineral density (BMD) and physical function in adults following MBS.

**Methods:**

Patients were randomized into aerobic, resistance, combined and control groups after undergoing MBS. Exercise groups completed a 26‐week supervised program (three sessions per week) with progressively increasing intensity. The primary outcome was the change in fat‐free mass (FFM) measured by dual‐energy X‐ray absorptiometry (DXA). Secondary outcomes included FM, hip, femoral neck and lumbar spine BMD, serum collagen type I C‐telopeptide (CTX) and physical function (e.g., 6‐min walk test and handgrip strength). Between‐group differences were assessed using Bayesian analysis of covariance (ANCOVA) adjusted for age, sex, surgery type and baseline body mass index, with credible differences inferred when the 95% credible interval of the standardized effect size excluded zero. Associations between outcomes were assessed using Pearson correlation.

**Results:**

Of 443 eligible candidates, 58 participants (aged 18–65 years, 70% women, BMI 41.7 ± 4.4 kg/m^2^) were randomized to aerobic (*n* = 15), resistance (*n* = 13), combined (*n* = 14) or control (*n* = 16) groups, and 91.3% were included in the analysis. The combined exercise group preserved more FFM (−5.1 ± 2.8 kg vs. −7.7 ± 2.8 kg; ES 0.60 [0.41, 0.77]) and reduced more FM (−11.9% ± 5.7% vs. −10.1% ± 4.9%, ES −0.64 [−0.85, −0.40]) than control. Aerobic training most attenuated total hip BMD loss (−0.037 ± 0.033 g/cm^2^, −3.3%) compared with control (−0.058 ± 0.021 g/cm^2^, −5.4%; ES 0.69 [0.42, 0.92]) and modestly attenuated femoral neck BMD loss (ES 0.30 [0.11, 0.48]), with no differences at the lumbar spine. The relative increase in serum CTX was smaller in the aerobic (157.8%; ES −0.44 [−0.65, −0.18]) and combined groups (161.8%; ES −0.42 [−0.63, −0.17]) compared with control (196.4%). Physical function improved modestly, with aerobic training showing a greater improvement in walking distance than control (80.0 ± 56.4 m vs. 65.6 ± 39.2 m; ES 0.33 [0.08, 0.54]). Walking distance improvement correlated with weight loss. Handgrip strength was best preserved in the resistance group (ES 0.77 [0.47, 1.04]) and was positively correlated with protein intake.

**Conclusions:**

Following MBS, combined aerobic and resistance training improved body composition by preserving FFM and reducing FM, while aerobic training most attenuated bone loss. Exercise was associated with modest improvements in physical function, supporting individualized exercise strategies to promote metabolic, musculoskeletal and functional health after MBS.

Trial Registration ClinicalTrials.gov Identifier: NCT04777305.

## 1 Introduction

Although the efficacy of metabolic bariatric surgery (MBS) in achieving substantial weight loss and improving obesity‐related comorbidities is well established [1, 2], it is also associated with substantial fat‐free mass (FFM) loss [3] (Data S1), particularly within the first 3–6 months postoperatively [3] (Data S1). These changes may adversely affect metabolic health and physical function [4]. In parallel, increased bone turnover and reductions in bone mineral density (BMD) occur during the first postoperative year, especially at weight‐bearing sites such as the hip and femoral neck joints [5, 6, 7].

Exercise is associated with fat mass (FM) reduction after MBS [8, 9, 10, 11, 12] (Data S2). Yet, its role in preserving FFM [8, 9, 11, 13, 14] (Data S2 and Data S3) and supporting sustained weight loss [9, 11] (Data S2, Data S3 and Data S4) remains unclear. Current postoperative guidelines recommend at least 150 min/week of moderate‐intensity aerobic exercise, combined with resistance training, targeting 300 min/week in total [15, 16]. However, these recommendations are derived from general population guidelines and may not adequately address the unique physiological challenges of MBS patients, including increased muscle catabolism, sedentary behaviour and sarcopenia risk [4, 17]. Adherence to these exercise recommendations among MBS patients is often poor [18, 19], highlighting the need for personalized, evidence‐based exercise strategies that consider physiological and lifestyle variations.

In this randomized controlled trial, we investigated the effects of structured aerobic, resistance and combined exercise interventions on the preservation of FFM following MBS. We also assessed the effects of these exercise regimens on FM and BMD changes and physical function.

## 2 Methods

The Physiological Outcomes with Exercise Regimens (POWER) bariatric randomized controlled trial was conducted between July 2022 and March 2024 at Tel Aviv University. The trial was approved by the ethics committees of Herzliya Medical Center (0010‐20‐HMC), Assuta Hospital (0056‐21‐ASMC) and Tel Aviv University (0003024‐6) and registered on ClinicalTrials.gov (NCT04777305) and MyTrial.gov (MOH_2021‐04‐22_009908). No commercial support was received. Participants were recruited from bariatric units at both medical centres and provided written informed consent before participation in the study.

### 2.1 Participants

Inclusion criteria comprised adults aged 18–65 years with obesity, defined as class I (BMI ≥ 35 kg/m^2^) [20], with at least one obesity‐related comorbidity, or Class II (BMI ≥ 40 kg/m^2^) [20] with or without obesity‐related comorbidities, who were candidates for primary MBS according to the National Institutes of Health (NIH) criteria [1, 21] and who had a sedentary lifestyle (not meeting minimum physical activity guidelines) [22]. Exclusion criteria comprised severe cardiopulmonary disease, musculoskeletal or neuromuscular impairments limiting physical activity, cognitive impairments, use of medications affecting bone or muscle metabolism, prior MBS or significant language barriers that may affect participants' ability to understand the study and exercise instructions. A sports medicine physician approved participants before the trial.

### 2.2 Randomization

Participants were randomly allocated into four groups consisting of three supervised exercise intervention groups: an aerobic exercise group, a resistance exercise group, a combined aerobic‐resistance exercise group and a control group that received standard bariatric care. Randomization followed a stratified block design with a 1:1:1:1 allocation ratio stratified by surgery type (sleeve gastrectomy (SG), Roux‐en‐Y gastric bypass (RYGB) or one‐anastomosis gastric bypass (OAGB)). A computer‐generated randomization scheme was used to ensure allocation concealment. Additional stratification by age, sex and BMI was applied to minimize potential confounding. Group allocation was open‐label for participants, study personnel and instructors, while data analysts were blinded to the group assignments. Each participant received a unique subject number, and personal data were securely stored with access limited to designated personnel.

### 2.3 Study Outcomes

The prespecified primary outcome was change in FFM (kg) and FM (%) was defined as the prespecified key secondary outcome. Additional prespecified secondary outcomes included changes in BMD, bone turnover marker and physical function, assessed at baseline and 26 weeks.

### 2.4 Intervention

**FIGURE 1:**
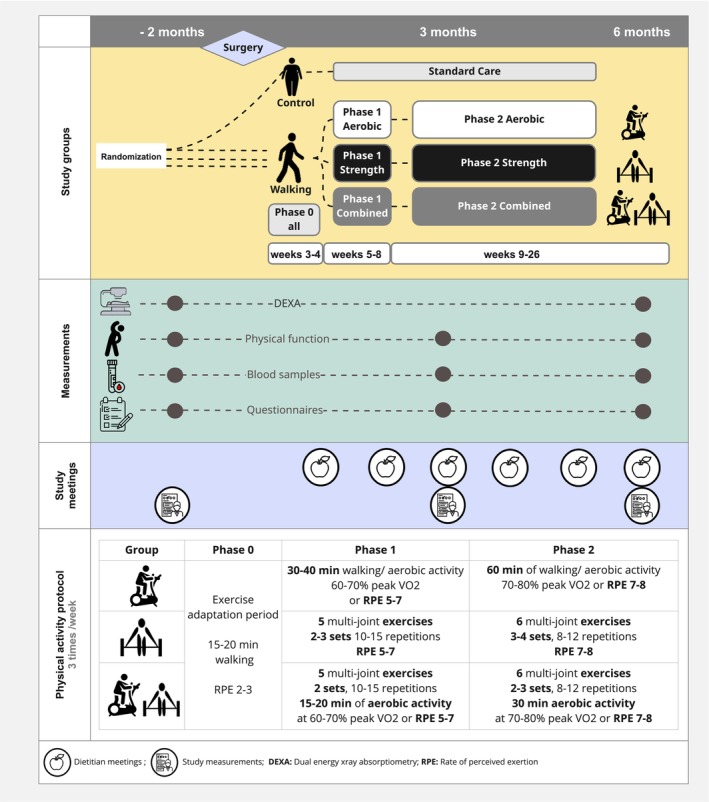
Study design and physical activity interventions.

The 26‐week intervention included three weekly exercise sessions, progressively increasing to 60 min in duration and intensity. Each participant was assigned a certified instructor to monitor their programme. At baseline, participants received equipment and live demonstrations before training. The programme progressed in phases (Figure 1, Data S1) beginning 3 weeks post‐MBS with a 2‐week adaptation phase (Phase 0, Figure 1) incorporating low‐intensity walking to promote early exercise habits while supporting postsurgical recovery. Each group then transitioned to structured, progressive training protocols (Data S1, Figure 1). Compliance was monitored weekly by the designated team to each participant, and adjustments were made in response to adherence challenges.

Resistance Training included multijoint strength exercises, progressing as participants adapted. Supervision occurred via Zoom, supplemented by video guides ( *YouTube channel* ). Participants received a set of universal free weights, incorporating both free‐weight and bodyweight exercises.

Aerobic Training consisted of moderate‐intensity activities such as walking, cycling or aerobic classes tracked with Polar Verity Sense‐VS heart rate monitors, linked to the Polar Flow app ( *Polar Flow* ). Monitors recorded training duration, frequency and heart rate in real time and were used to ensure weekly goals were met.

Combined Exercise integrated both aerobic and resistance training, progressively increasing in complexity. Participants received both heart rate monitors and universal free weights.

Adherence to exercise intervention was defined as completing > 70% of the prescribed sessions. For resistance training, adherence was determined based on attendance at supervised sessions, whereas for aerobic training, adherence was verified using Polar heart rate‐recorded sessions.

Control Group received standard bariatric care.

#### 2.4.1 Standard Postoperative Care

All four groups received monthly group sessions with a registered dietitian, covering bariatric diet progression, balanced diet aligned with bariatric guidelines [16, 23], protein intake of ≥ 60 g/day (or 1.0–1.5 g/kg ideal body weight) or up to 90–100 g/day (2.1 g/kg ideal body weight) for malabsorptive procedures [16, 23, 24], mindful eating, food environment, barriers and lifestyle planning. These sessions did not replace participants' individual nutritional follow‐up provided by their health care providers, who oversaw postoperative supplementation and monitoring according to national and international bariatric guidelines.

### 2.5 Outcomes

#### 2.5.1 Body Composition and BMD

FFM, FM and BMD were assessed at baseline and 26 weeks using dual‐energy X‐ray absorptiometry (DXA, Lunar Prodigy; GE Healthcare), calibrated daily. Participants were positioned centrally, hands midprone and feet secured with a Velcro strap (Data S5), BMD was measured at the total hip, femoral neck and lumbar spine. Scans were performed at the same time of day for each participant, following a ≥ 4‐h fasting period, ≥ 48 h without exercise and ≥ 24 h without intake of calcium‐containing supplements (including multivitamins and antacids such as TUMS). If participants had recently undergone imaging with barium, CT contrast or radioisotopes, DXA assessments were postponed for 10 to 14 days. Visual inspection ensured consistency with baseline scans. Positioning errors were corrected using the rescan function, and a mirror scanning technique was employed for body composition for individuals whose body dimensions exceeded the DXA frame.

#### 2.5.2 Anthropometric Measurements

Weight was measured to the nearest 0.1 kg using a digital scale (SECA mBCA 515; SECA, Hamburg, Germany) and height to the nearest 0.5 cm using a SECA 274 Free‐Standing Wireless Stadiometer. Waist circumference was measured twice at each site (umbilicus and at superior border of the iliac crest according to NIH criteria [25]). BMI and percentage of excess weight loss (EWL%) were calculated with ideal body weight calculated at a BMI of 25 kg/m^2^.

$$\mathrm{EWL}\%\text{calculation}=\frac{\left[\left(\text{initial weight}\right)-\left(\text{post operative weight}\right)\right]}{\left[\left(\text{initial weight}\right)-\left(\text{ideal body weight}*\right)\right]}*100$$

#### 2.5.3 Physical Function

Assessment included standardized scripts and demonstrations, without verbal encouragement for consistency.

Handgrip strength was assessed using a *Baseline* Hydraulic Standard Hand Dynamometer (200‐lb capacity). Participants sat on an armless chair with the elbow extended, feet flat and hips and knees flexed at 90°. After familiarization, two maximal grip efforts were performed by the dominant hand, and the average value was calculated.

The 6‐min walk test assessed the maximum distance covered in 6 min on a flat track. The Sit‐to‐Stand Test followed a practice repetition, with participants completing five stand–sit cycles as quickly as possible.

Aerobic fitness measured by submaximal graded cycle ergometer test, targeting 80% of predicted maximal heart rate, measured by indirect calorimetry (Quark CPE, COSMED, Rome, Italy).

Muscle strength was assessed using the one‐repetition maximum (1RM) test for chest and leg press, following a no‐weight familiarization, submaximal attempts and up to six 1RM attempts.

#### 2.5.4 Nutritional Intake

The *MealLogger* app using a 3‐day food diary at baseline and follow‐up was used for the assessment. Participants recorded meals with descriptions and photos. Intake was analysed in NutRatio, a database suited for the Israeli population, integrating USDA (SR‐28, 2018) (Data S6) and Israeli Ministry of Health (2017) databases (Data S7).

#### 2.5.5 Daily Physical Activity

Daily activity tracking was through wrist‐worn accelerometers (Actigraph GT9X) on the nondominant wrist for > 4 days at both baseline (*n* = 36) and follow‐up (*n* = 21). Exercise compliance was assessed through (1) supervised resistance training session attendance during 22 weeks of supervised training and (2) aerobic session tracking via heart rate monitor, synced with Polar Flow app, alongside self‐reported activity type, duration and intensity.

#### 2.5.6 Blood Sampling and Analysis

Fasting blood samples (20 mL) were collected in the morning after an overnight fast of ≥ 12 h, following a 15‐min equilibration period, at baseline and after 26 weeks. Samples were centrifuged to separate serum, which was aliquoted and stored at −80°C until analysis. CTX concentrations were determined using an automated electrochemiluminescence system (E411; Roche Diagnostics, Mannheim, Germany).

### 2.6 Statistical Analysis

The POWER trial was originally designed with frequentist statistics. A difference of 5 ± 6.7 kg in FFM between groups was considered clinically meaningful [26] and accordingly, the minimal sample size per group was 13 patients assuming an 80% power, alpha of 0.05 and 25% dropout rate. Categorical variables were reported as counts and percentages, and continuous variables as mean ± SD (and median with IQR when appropriate). Pearson correlation assessed associations between BMD, body weight and composition, physical function and dietary intake using SPSS 29 (SPSS Inc., Chicago, IL, USA).

A modified Intention‐to‐treat (mITT) analysis included all randomized participants who completed baseline and at least one additional postbaseline assessment. Missing follow‐up data within the mITT population were assumed to be missing at random and were handled using multiple imputation (Data S8). In line with the American Statistical Association's recommendations to no longer present analyses using *p*‐values [27], (Data S9) between‐group differences in changes from baseline to 6‐month postsurgery were analysed using Bayesian (Data S10, Data S11 and Data S12) ANCOVA on multiply imputed datasets (*m* = 5, predictive mean matching), adjusting for age, sex, surgery type and baseline BMI. Models were fitted via brms (Data S13 and Data S14) with weakly informative priors: normal (0,10) for fixed effects (including intercept) and exponential (1) for residual standard deviation. Markov Chain Monte Carlo (MCMC) sampling was implemented with four chains, 2000 iterations per chain (including 1000 warmup iterations for stabilization) and an adapt delta parameter of 0.95 to enhance convergence and reduce divergences. Model fit was assessed via posterior predictive checks (implicit in brms diagnostics), effective sample sizes (> 400 per parameter) and Gelman–Rubin R‐hat values (< 1.01), ensuring reliable posterior inference. Posterior distributions yielded standardized effect sizes (ES; mean and 95% credible intervals) relative to the Control group. Credible differences were inferred when the 95% credible interval of the effect size excluded zero.

All Bayesian analyses were conducted using R version 4.4.2 (R Foudation for Statistical Computing, Vienna, Austria).

## 3 Results

**FIGURE 2:**
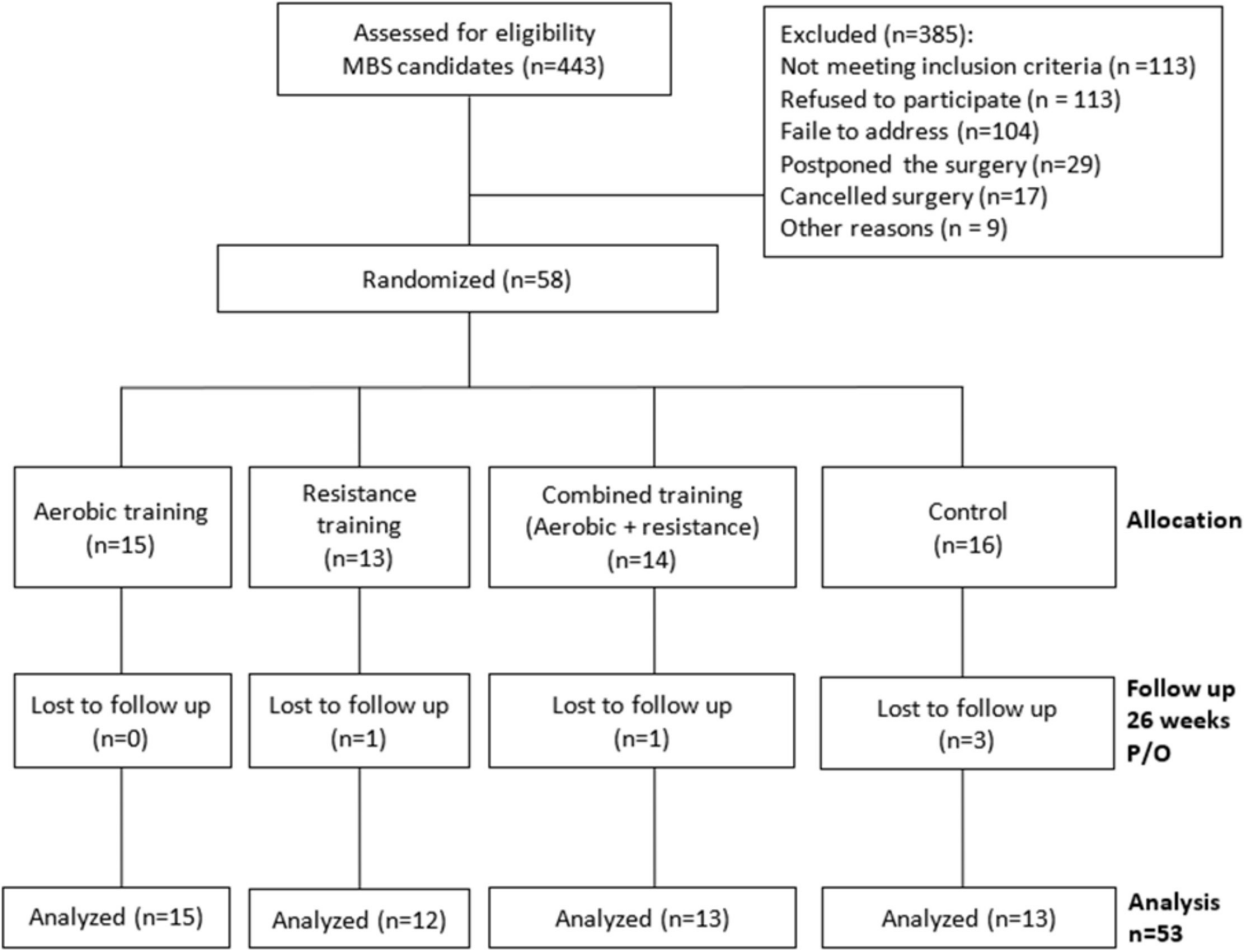
POWER bariatric randomized controlled trial study flow diagram. Abbreviations: MBS, metabolic bariatric surgery; P/O, postoperative. Exclusion criteria: Severe cardiopulmonary disease, musculoskeletal or neuromuscular impairments, cognitive impairments, use of drugs affecting bone or muscle metabolism, previous MBS and major language barriers.

Of 443 eligible candidates, 58 were randomized (aged 18–65 years, 70% women, BMI 41.7 ± 4.4 kg/m^2^). A total of 53 participants (91.3%) completed at least one postbaseline assessment and were included in the modified ITT analytic sample: 15 in the aerobic group, 12 in the resistance group, 13 in the combined group and 13 in the control group (Figure 2, Table 1).

**TABLE 1:** Baseline characteristics of study population.

	Aerobic *N* = 15	Resistance *N* = 13	Combined *N* = 14	Control *N* = 16
Age (years)	39.2 ± 10.1 (39.5)	41.0 ± 9.3 (42.5)	38.7 ± 12.0 (36.2)	38.0 ± 10.6 (39.0)
Female *N* (%)	12 (80.0)	8 (66.7)	10 (76.9)	10 (76.9)
Anthropometric and body composition				
Weight (kg)	114.8 ± 17.1 (117.2)	117.5 ± 20.6 (109.4)	110.1 ± 17.2 (105.0)	116.2 ± 15.2 (113.9)
BMI (kg/m^2^)	41.9 ± 3.7 (41.4)	42.1 ± 3.9 (42.6)	40.7 ± 5.5 (39.2)	42.3 ± 4.7 (41.5)
Waist circumference (cm)	123.8 ± 11.3 (123.1)	129.0 ± 14.4 (128.3)	120.4 ± 11.9 (115.9)	127.6 ± 11.6 (124.3)
Hip circumference (cm)	125.6 ± 12.3 (127.1)	130.2 ± 15.1 (128.8)	123.8 ± 11.7 (125.1)	128.1 ± 11.8 (125.5)
Fat mass %	50.5 ± 6.8 (53.0)	48.8 ± 4.7 (49.6)	50.8 ± 5.7 (51.7)	50.3 ± 5.7 (52.3)
FFM (kg)	56.6 ± 13.6 (52.8)	59.3 ± 12.2 (53.2)	52.8 ± 8.2 (53.7)	57.5 ± 12.4 (53.3)
Surgery type				
RYGB, *N* (%)	3 (20.0)	2 (16.7)	2 (15.4)	6 (46.2)
SG, *N* (%)	7 (46.7)	2 (16.7)	2 (15.4)	3 (23.1)
OAGB, *N* (%)	5 (33.3)	8 (66.7)	9 (69.2)	4 (30.8)
Bone mineral density (g/cm^2^)				
Total hip	1.116 ± 0.129	1.194 ± 0.172	1.141 ± 0.087	1.091 ± 0.157
Femoral neck	1.078 ± 0.111	1.153 ± 0.235	1.098 ± 0.079	1.049 ± 0.141
Lumbar spine	1.298 ± 0.176	1.314 ± 0.208	1.309 ± 0.132	1.249 ± 0.262
Serum CTX (ng/mL)	0.346 ± 0.129	0.279 ± 0.108	0.325 ± 0.092	0.268 ± 0.077
Menopause *N* (%)*	**2 (13.3)**	**1 (7.6)**	**2 (14.2)**	**0 (0)**
Physical fitness and strength				
1RM leg (kg)	119.9 ± 35.8 (117.3)	125.8 ± 31.5 (122.6)	121.0 ± 42.1 (116.0)	112.7 ± 38.0 (109.0)
1RM leg/body weight (kg)	1.0 ± 0.3 (1.1)	1.1 ± 0.2 (1.2)	1.1 ± 0.4 (1.1)	1.0 ± 0.3 (1.1)
1RM chest (kg)	36.6 ± 18.0 (30.0)	42.4 ± 24.4 (36.3)	35.3 ± 18.0 (26.3)	33.3 ± 21.8 (25.0)
1RM chest/body weight (kg)	0.3 ± 0.1 (0.3)	0.4 ± 0.2 (0.3)	0.3 ± 0.2 (0.3)	0.3 ± 0.2 (0.2)
Hand grip (kg)	29.4 ± 11.8 (24.5)	31.4 ± 12.7 (28.5)	32.3 ± 10.1 (29.0)	31.7 ± 13.8 (26.1)
Sit to stand (seconds)	7.9 ± 2.0 (7.9)	7.5 ± 1.7 (6.8)	7.5 ± 2.0 (7.0)	9.0 ± 2.5 (9.7)
6‐Min walk (m)	473.7 ± 63.1 (461.7)	498.1 ± 38.5 (504.5)	495.5 ± 74.7 (498.6)	445.9 ± 49.0 (459.9)
Vo2 at 80% Max (mL/min)	1922 ± 595 (1945)	1882 ± 312 (1837)	1796 ± 365 (1725)	1740 ± 563 (1696)
Vo2/kg at 80% Max (mL/kg/min)	16.6 ± 3.6 (15.6)	16.1 ± 1.6 (16.1)	16.5 ± 3.2 (16.9)	14.9 ± 3.1 (15.5)
Vo2/FFM at 80% Max (mL/kg FFM/min)	34.0 ± 7.1 (34.1)	31.7 ± 3.6 (31.3)	33.5 ± 5.0 (32.9)	29.4 ± 6.2 (29.3)
Watt at 80% max (Watt/min)	118.4 ± 34.1 (110.0)	112.2 ± 33.5 (109.0)	111.1 ± 34.2 (107.5)	115.1 ± 39.2 (116.0)
Daily diet composition				
Total energy intake (Kcal)	1102 ± 429 (1070)	1615 ± 716 (1507)	1239 ± 245 (1320)	1311 ± 351 (1386)
Carbohydrates (%)	28.1 ± 7.8 (26.5)	35.7 ± 10.0 (39.6)	37.1 ± 14.2 (34.9)	34.6 ± 10.5 (39.1)
Protein (%)	27.1 ± 5.1 (27.0)	25.1 ± 6.0 (25.5)	24.5 ± 7.4 (23.2)	24.2 ± 5.1 (25.1)
Lipid (%)	44.6 ± 5.6 (45.8)	38.0 ± 6.7 (39.6)	37.7 ± 9.7 (38.4)	41.1 ± 8.6 (41.2)
Physical activity habits				
Frequency of trainings (times per week)	1.4 ± 1.4 (1.0)	0.3 ± 0.5 (0.0)	1.0 ± 1.3 (0.0)	0.7 ± 0.9 (0.0)
Total weekly training (min/week)	53.6 ± 68.4 (45.0)	18.6 ± 30.2 (0.0)	51.7 ± 67.6 (0.0)	22.1 ± 37.9 (0.0)
RPE average	3.3 ± 2.7 (3.0)	1.6 ± 2.1 (1.0)	3.0 ± 3.1 (2.5)	2.3 ± 2.4 (3.0)

*Note:* Submaximal VO_2_ was assessed during a graded ergometer biking test. Total one‐repetition maximum (1RM) is the total of the maximum weight a participant can lift, in one attempt, in the chest press and leg press. Numerical variables are presented as mean ± standard deviation (median), and categorical variables as *n* (%). Table includes all randomized participants. * Percentages are based on the full randomized sample.

Abbreviations: 1RM, one‐repetition maximum; BMI, body mass index; CTX, C‐terminal telopeptide of type I collagen; FFM, fat‐free mass; Kcal, kilocalories; OAGB, one‐anastomosis gastric bypass; RPE, rating of perceived exertion at baseline on a 1–10 Borg scale; RYGB, Roux‐en‐Y gastric bypass; SG, sleeve gastrectomy; VO_2_ at 80% max, volume of oxygen consumption (mL/min) at 80% of maximal heart rate predicted.

### 3.1 Adherence to Exercise Intervention

Training duration and intensity increased from 41.6 ± 59.1 min/week at baseline, with a mean of 2.7 ± 2.7 on the Borg Rating of Perceived Exertion (RPE) scale (1 to 10), to 92.1 ± 67.3 min/week, and an RPE of 5.8 ± 2.1 at 6‐month follow‐up in the intervention groups. Participants in the training groups attended on average 62.9% of the supervised exercise sessions over 26 weeks after MBS (22 weeks of the designated exercise regimen). Accelerometer monitoring showed that participants spent 53.0% ± 6.4% of the day sedentary or sleeping, 34.9% ± 4.3% in light activity, and 12.0% ± 3.4% in moderate activity.

### 3.2 Weight and Body Composition Changes

**FIGURE 3:**
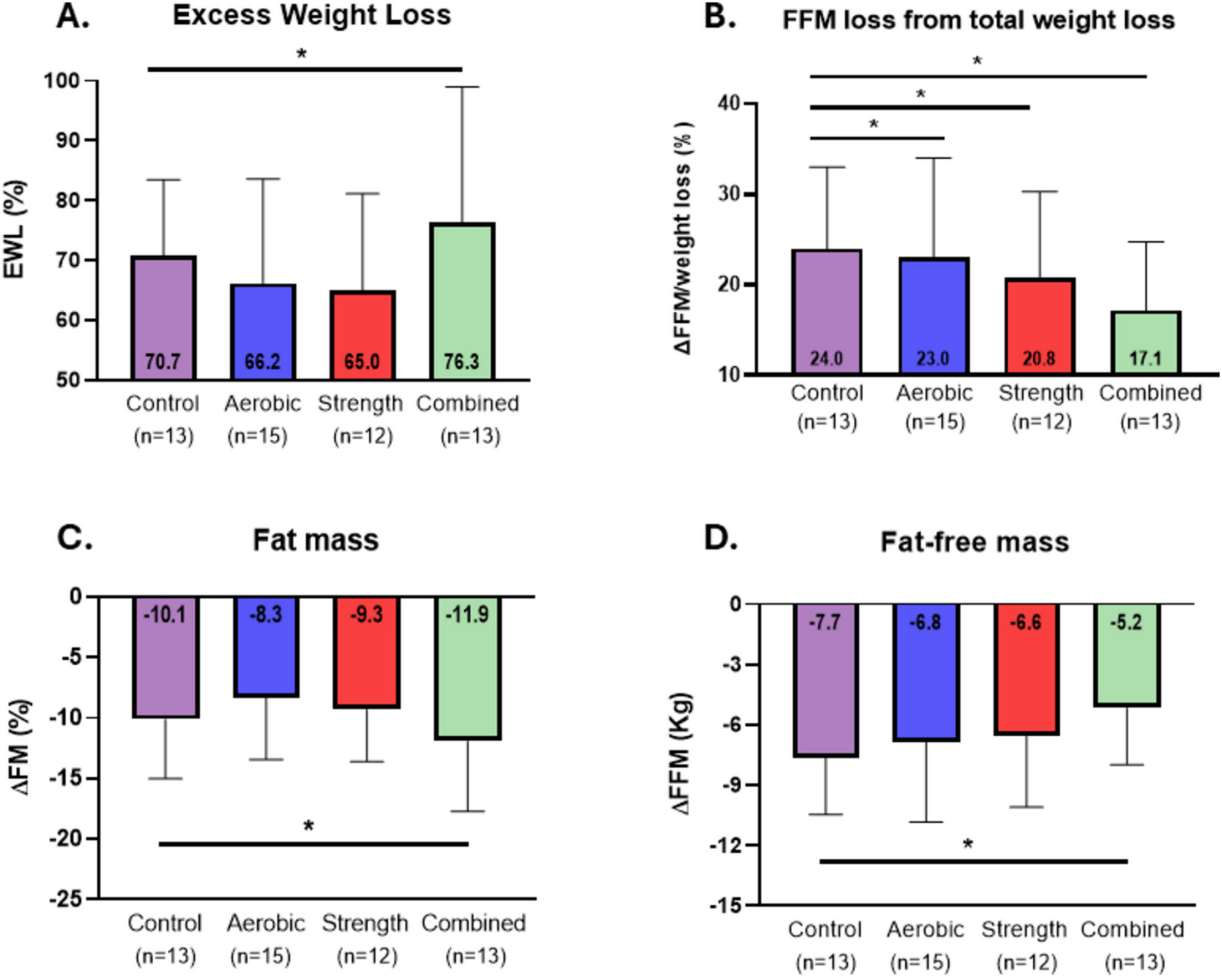
Changes in body composition and weight loss (A–D) Bars show mean ± SD differences between exercise and control were estimated using Bayesian ANCOVA adjusted for age, sex, surgery type and baseline BMI. Credible differences were inferred when the adjusted 95% credible interval of the standardized effect size excluded zero, and the effect size exceeded a negligible magnitude (|ES| > 0.2) and are indicated by an asterisk (*). Abbreviations: EWL: excess weight loss; FFM: fat‐free mass; FM: fat mass.

Participants lost 31.0 ± 8.3 kg corresponding to a total weight loss percentage (TWL%) of 26.9% ± 5.6% and EWL% of 69.4% ± 17.5%. The combined exercise group achieved a greater EWL% (76.3% ± 22.7%), compared with the control group (70.7% ± 12.8%, ES 0.60 [0.35, 0.81], Table 2, Figure 3A) and achieved the greatest improvement in body composition, including FM reduction of −11.9% ± 5.7%; ES −0.64 [−0.85, −0.40] and greater FFM preservation of −5.1 ± 2.8 kg; ES 0.60 [0.41, 0.77], compared with the control group (Table 2, Figure 3C,D). Both the resistance (20.8% ± 9.6%) and combined (17.4% ± 7.6%) groups preserved a greater proportion of FFM relative to total weight loss compared with the control group (24.0% ± 9.0%, Table 2, Figure 3B) with a smaller but credible effect also observed in the aerobic group (ES −0.26).

**TABLE 2:** Changes in anthropometric, body composition, bone and physical function outcomes by intervention group.

	Aerobic (mean ± SD) *N* = 15	Resistance (mean + SD) *N* = 12	Combined (mean + SD) *N* = 13	Control (mean + SD) N = 13	Effect size mean ±95% credible interval: aerobic vs. control	Effect size mean ±95% credible interval: resistance vs. control	Effect size mean ±95% credible interval: combined vs. control
Weight and body composition							
Δ Weight (kg)	−29.6 ± 7.0	−30.4 ± 9.4	−31.9 ± 10.6	−32.5 ± 7.0	0.36 [0.19, 0.52]*	0.18 [−0.03, 0.39]	0.26 [−0.01, 0.51]
EWL%	66.2 ± 17.4	65.0 ± 16.1	76.3 ± 22.7	70.7 ± 12.8	−0.16 [−0.31, 0.01]	−0.09 [−0.30, 0.12]	0.60 [0.35, 0.81]*
ΔFFM (kg)	−6.8 ± 4.0	−6.6 ± 3.5	−5.1 ± 2.8	−7.7 ± 2.8	0.07 [−0.14, 0.28]	0.01 [−0.22, 0.25]	0.60 [0.41, 0.77]*
ΔFM (%)	−8.3 ± 5.1	−9.3 ± 4.3	−11.9 ± 5.7	−10.1 ± 4.9	0.16 [−0.03, 0.35]	0.04 [−0.16, 0.22]	−0.64 [−0.85, −0.40]*
ΔFFM/weight loss (%)	23.0 ± 11.0	20.8 ± 9.6	17.4 ± 7.6	24.0 ± 9.0	−0.26 [−0.47, −0.04]*	−0.52 [−0.72, −0.28]*	−0.98 [−1.17, −0.72]*
Bone measures							
Total hip (g/cm^2^)	−0.037 ± 0.033	−0.056 ± 0.035	−0.052 ± 0.033	−0.058 ± 0.021	0.69 [0.42, 0.92]*	0.08 [−0.18, 0.33]	0.20 [−0.04, 0.43]
∆ Relative (%)	−3.3 ± 2.8	−4.7 ± 2.8	−4.6 ± 2.9	−5.4 ± 1.9	0.82 [0.54, 1.05]*	0.26 [0.01, 0.50]*	0.32 [0.06, 0.56]*
Femoral neck (g/cm^2^)	−0.033 ± 0.032	−0.049 ± 0.067	−0.043 ± 0.042	−0.046 ± 0.032	0.30 [0.11, 0.48]*	−0.07 [−0.43, 0.30]	0.06 [−0.15, 0.28]
∆ Relative (%)	−3.2 ± 3.0	−3.9 ± 5.8	−3.9 ± 3.7	−4.3 ± 2.9	0.32 [0.12, 0.50]*	0.08 [−0.28, 0.44]	0.12 [−0.10, 0.34]
Lumbar spine (g/cm^2^)	−0.014 ± 0.044	−0.003 ± 0.046	−0.009 ± 0.055	−0.010 ± 0.035	−0.10 [−0.32, 0.13]	0.15 [−0.09, 0.39]	0.06 [−0.23, 0.35]
∆ Relative (%)	−1.1 ± 3.6	−0.3 ± 3.6	−0.7 ± 4.1	−0.8 ± 2.9	−0.09 [−0.32, 0.15]	0.13 [−0.12, 0.38]	0.02 [−0.25, −0.31]*
Serum CTX (ng/mL)	0.482 ± 0.211	0.528 ± 0.255	0.512 ± 0.314	0.501 ± 0.184	−0.08 [−0.29, 0.13]	0.18 [−0.12, 0.45]	0.05 [−0.25, 0.35]
∆ Relative (%)	157.8 ± 92.4	212.7 ± 110.7	161.8 ± 88.1	196.4 ± 70.4	−0.44 [−0.65, −0.18]*	0.17 [−0.12, 0.45]	−0.42 [−0.63, −0.17]*
Physical function							
∆1RM Leg (kg)	−22.0 ± 32.8	−8.9 ± 29.3	−21.8 ± 44.4	−8.4 ± 33.8	−0.53 [−0.71, −0.31]*	−0.06 [−0.26, 0.14]	−0.42 [−0.63, −0.18]*
∆1RM leg/body weight (kg)	0.1 ± 0.4	0.3 ± 0.3	0.1 ± 0.4	0.3 ± 0.4	−0.67 [−0.85, −0.44]*	−0.14 [−0.31, 0.05]	−0.61 [−0.83, −0.36]*
∆Hand grip (kg)	−0.6 ± 4.2	1.5 ± 6.1	−1.2 ± 3.6	−2.2 ± 5.6	0.38 [0.17, 0.56]*	0.77 [0.47, 1.04]*	0.35 [0.14, 0.53]*
∆Sit to stand (seconds)	−0.21 ± 2.22	−0.50 ± 1.71	−0.60 ± 1.51	−1.39 ± 1.70	0.64 [0.37, 0.88]*	0.56 [0.32, 0.76]*	0.55 [0.37, 0.71]*
∆6‐Min walk (m)	80.0 ± 56.4	52.8 ± 40.5	58.7 ± 36.7	65.6 ± 39.2	0.33 [0.08, 0.54]*	−0.26 [−0.46, −0.04]*	−0.24 [−0.39, −0.06]*
ΔVo2/kg at 80% max (mL/kg/min)	3.3 ± 3.5	3.6 ± 3.0	4.9 ± 3.8	3.4 ± 4.2	−0.32 [−0.52, −0.09]*	−0.51 [−0.71, −0.28]*	−0.15 [−0.36, −0.07]*
ΔVo2/FFM at 80% Max (mL/kg FFM/min)	0.2 ± 7.0	0.8 ± 4.6	1.9 ± 4.4	1.1 ± 7.8	0.06 [−0.17, 0.28]	0.04 [−0.26, 0.19]	−0.01 [−0.22, 0.20]
ΔWatt at 80% max (Watt/min)	5.4 ± 20.1	8.3 ± 17.6	3.8 ± 19.7	−5.8 ± 20.6	0.44 [0.23, 0.62]*	0.82 [0.59, 1.02]*	0.53 [0.31, 0.72]*

*Note:* Submaximal VO_2_ was assessed during a graded ergometer biking test. Numerical variables are presented as observed (unadjusted) mean ± standard deviation. Six‐month changes from baseline are represented by Δ (delta). Negative values represent decreases, and positive values indicate increases in the parameters. Comparisons between each exercise group versus control are presented as standardized effect sizes with 95% credible intervals [lower—upper bound] derived from Bayesian ANCOVA, controlling for age, sex, surgery type and baseline BMI. Effect sizes were considered to indicate credible differences when the 95% credible interval did not include zero. These are indicated in bold and marked with an asterisk (*). The table includes only participants who contributed data to the respective analyses.

Abbreviations: 1RM, one‐repetition maximum; BMD, bone mineral density; CTX, C‐terminal telopeptide of type I collagen; EWL%, excess weight loss%; FM, fat mass; FFM, fat‐free mass.

### 3.3 Bone Mineral Density

Greater dietary protein intake (g/day) at baseline was positively correlated with total hip BMD (*r* = 0.41, *p* = 0.015) and femoral neck BMD (*r* = 0.51, *p* = 0.002). FFM (kg) also positively correlated with total hip (*r* = 0.33, *p* = 0.021) and femoral neck BMD (*r* = 0.32, *p* = 0.029).

**FIGURE 4:**
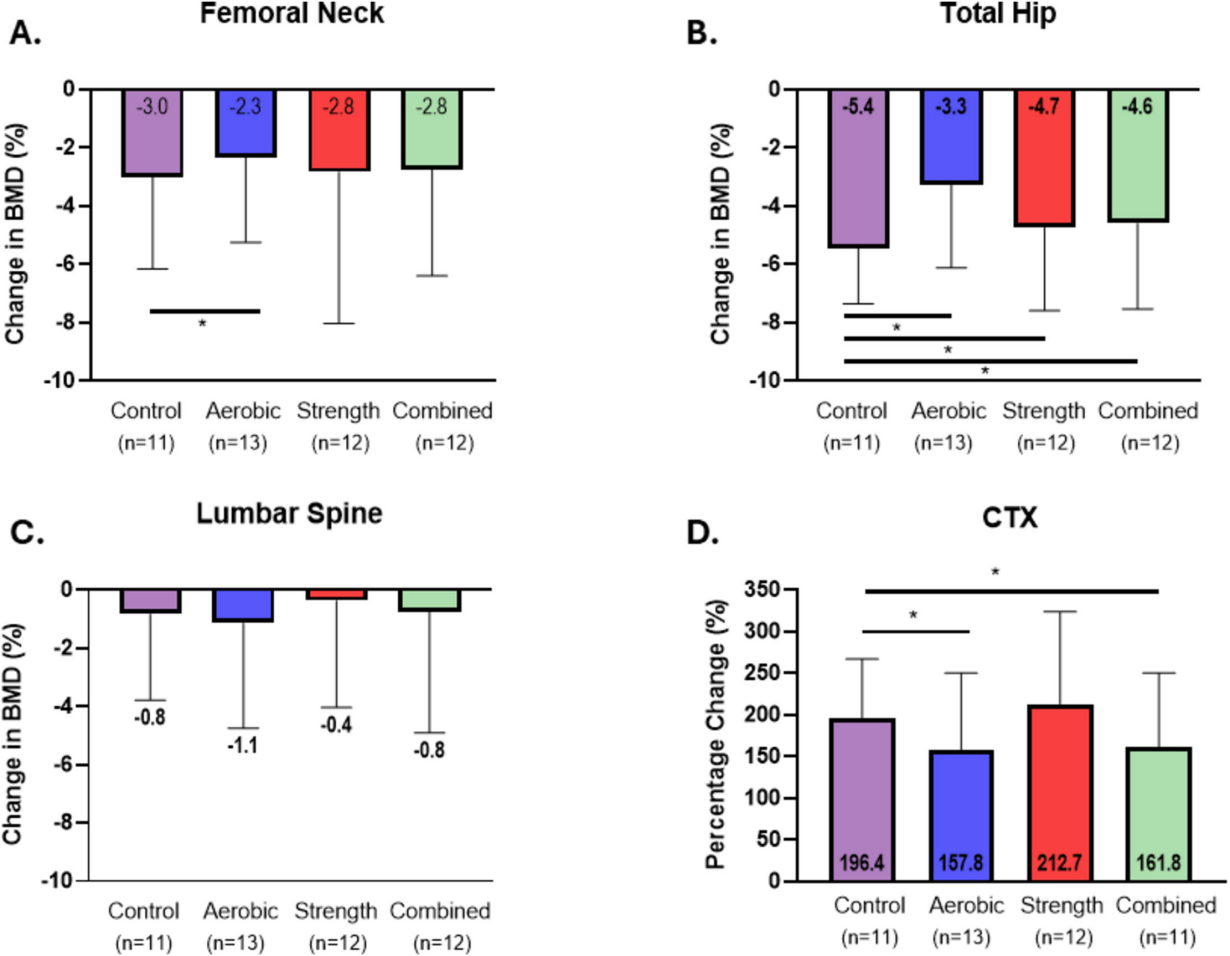
Changes in bone mineral density and bone marker. (A–D) Bars show mean ± SD differences between exercise and control were estimated using Bayesian ANCOVA adjusted for age, sex, surgery type and baseline BMI. Credible differences were inferred when the adjusted 95% credible interval of the standardized effect size excluded zero, and the effect size exceeded a negligible magnitude (|ES| > 0.2) and are indicated by an asterisk (*). Abbreviations: BMD, bone mineral density; CTX, serum collagen type I C‐telopeptide.

The decline in total hip BMD was attenuated in the aerobic group (−0.037 ± 0.033 g/cm^2^, −3.3%) compared with the control group (−0.058 ± 0.021 g/cm^2^, −5.4%, ES 0.69) with smaller but credible differences also observed only for relative total hip BMD in the resistance (−4.7%, ES 0.26) and combined groups (−4.6%, ES 0.32, Table 2, Figure 4). At the femoral neck, aerobic training was associated with a modest but credible attenuation of BMD loss (ES 0.30), whereas resistance and combined training did not differ from control (Table 2, Figure 4). No credible between‐group differences were observed at the lumbar spine. Serum CTX increased less in the aerobic (157.8%, ES −0.44) and combined (161.8%, ES −0.42) groups than in the control group (196.4%, Table 2), with no credible difference observed for the resistance group. Changes in total hip BMD were significantly correlated with changes in body weight (*r* = 0.50, *p* < 0.001), FM% (*r* = 0.45, *p* = 0.001), EWL% (*r* = −0.47, *p* = 0.001) and serum CTX (*r* = −0.46, *p* = 0.001). Vitamin supplementation during follow‐up was reported by 49 participants, of whom 95.7% consumed a multivitamin that typically included calcium and vitamin D, and 85.6% took additional vitamin D. Additional supplements, which were not routinely required, included iron (27.5%) and vitamin B12 (32.1%).

### 3.4 Physical Function

Handgrip strength increased only in the resistance group (1.5 ± 6.1 kg vs. −2.2 ± 5.6 kg in controls, ES 0.77), with the greatest decline observed in controls compared with all intervention groups (Table 2). The 6‐min walk improved across all groups. Aerobic training was associated with a modest but credible greater improvement compared with control (80.0 ± 56.4 m vs. 65.6 ± 39.2 m; ES 0.33, Table 2), whereas differences for the resistance and combined groups were small in magnitude. Walking distance gains correlated with weight changes, meaning that those with greater weight loss had a greater improvement in walking distance (*r* = −0.291, *p* = 0.037). Absolute lower body strength declined across all groups. Relative strength (1RM/body weight) was largely preserved; however, no credible advantage was observed for the exercise groups compared with control, with aerobic and combined training showing slightly less favourable changes. We found greater improvements among participants who met postoperative protein intake targets (Data S2). In contrast, baseline protein intake was not related to preservation of FFM or strength. Sit‐to‐stand test improved most in controls, trending for a negative correlation with older age (*r* = −0.254, *p* = 0.070) and a negative correlation with caloric intake at follow‐up, showing an improvement as the age and caloric intake increased (*r* = −0.346, *p* = 0.031). At 80% of maximal capacity, oxygen uptake per kg of bodyweight showed less favourable changes in the resistance group (3.6 ± 3.0 vs. 3.4 ± 4.2 mL/kg/min in controls; ES −0.51), with smaller negative effects in the aerobic and combined groups (Table 2), whereas all exercise groups demonstrated greater improvements in power output, most pronounced in the resistance group (8.3 ± 17.6 vs. −5.8 ± 20.6 Watt/min; ES 0.82).

### 3.5 Adverse Events

Four adverse events occurred, none related to exercise: one case of cholelithiasis requiring cholecystectomy, two minor motor vehicle accidents and one ovarian cyst.

## 4 Discussion

This study evaluated the effects of exercise regimens on body composition, BMD and physical function of 6 months post‐MBS. All groups improved, with the combined exercise group demonstrating the greatest benefits in %EWL, FFM preservation and FM reduction, and the aerobic group showed the better BMD preservation, especially in the total hip and femoral neck. Physical function improved in all groups, whereas absolute strength declined mostly in the aerobic and combined groups; however, relative strength was maintained. Handgrip strength was better preserved in the exercise groups, with the resistance group showing the most pronounced effect. These findings suggest that combined training is an effective approach for preserving FFM and improving body composition during the early post‐MBS period.

In our study, combined training was the most effective intervention for FFM preservation, accounting for 17.4% of total body weight loss 6 months post‐MBS. A 3‐year study (*n* = 3596) reported FFM loss of 23% at 3 months, decreasing to 21.3% at 6 months, but rising to 24.7% at 36 months, highlighting the long‐term impact of body composition changes [3]. Moreover, a review and meta‐regression showed that FFM and FM loss contribute more to weight regain variability than weight loss alone [28] and FFM loss has been associated with sarcopenia [29], particularly when accompanied by low strength and reduced muscle quality often observed in obesity [29, 30]. Despite exercise's potential, evidence for its effectiveness in preserving FFM after MBS remains inconsistent [8, 9, 11, 13, 14] (Data S2 and Data S3). Our findings support incorporating combined aerobic‐resistance training to preserve FFM in the early phase after MBS.

Although BMD was assessed relatively early postoperatively, we found significant changes. The aerobic training group showed greater preservation of total hip BMD and femoral neck compared to controls, accompanied by smaller increases in serum CTX levels, which were also found in the combined group indicating reduced bone resorption. These findings suggest that aerobic training may help attenuate increases in bone turnover in the rapid weight‐loss phase, although the mechanisms underlying this effect remain unclear. These findings support previous randomized controlled trials showing that structured exercise can attenuate postbariatric BMD loss at multiple skeletal sites [31, 32] (Data S15) and align with evidence that combined aerobic and resistance training have a protective effect on BMD after RYGB. Additionally, combining exercise, vitamin D, calcium and BMI‐adjusted protein intake further slowed BMD loss [33] and combining resistance, walking and high‐impact activities enhanced bone preservation [31]. Aerobic exercise may be particularly effective due to the skeletal loading imposed by excess body weight during weight‐bearing activity, potentially exceeding the load applied in resistance training, especially in the early phase after MBS, when weight load is usually high. Thus, aerobic modalities may offer a practical strategy for supporting bone health during the early postoperative period. Moreover, positive correlations between postoperative protein intake, FFM and BMD highlight the importance of adequate nutritional support during the early postoperative period, when restricted intake may compromise muscle strength and physical function. These results are consistent with prior studies showing that higher protein intake (particularly from animal sources) and calcium supplementation (800 mg) are associated with improved BMD and reduced hip fracture risk [34] (Data S16) and support the importance of lean mass preservation in mitigating postsurgical BMD loss [35]. To better reflect a real‐world postoperative setting, we advised participants not to combine multiple nutritional supplements, such as protein shakes and instead to prioritize obtaining protein from food. This approach minimized potential bias and allowed clearer evaluation of the direct effects of exercise on body composition and metabolic outcomes.

Physical function improvements varied. Walking distance improved across groups, with a modest advantage for aerobic training over control, and also likely improved due to weight and FM loss, enhancing range of motion and gait [4], whereas oxygen uptake normalized to body weight did not show favourable changes. Despite absolute strength loss, relative strength was preserved, aligning with prior research [5, 36]. Handgrip strength was highest in the resistance group and improved across all intervention groups relative to controls, suggesting a beneficial effect of exercise on early postoperative muscle function. Although some studies report strength gains within the first 6‐month post‐MBS [13], ongoing physical activity monitoring remains crucial. Exercise benefits may become clearer as weight loss stabilizes [14, 37] (Data S17). Combining resistance training with protein supplementation early after MBS may further enhance strength [38], potentially reducing weight regain risk even after training cessation [39].

### 4.1 Limitations and Strengths

The main limitation of this study is the sample size, which may have reduced sensitivity to detect subtle differences between exercise groups. Additional limitations may be mainly related to the free‐living design of our study, where variability in adherence to the study could have diluted or added variability to the effects, although this was partially addressed using accelerometers and activity questionnaires. Additionally, the 6‐month follow‐up may have been too early to detect more meaningful BMD changes, which are typically assessed at 1 year or later; in this context, CTX may have provided earlier insight into bone turnover dynamics. Menopausal status could not be adequately accounted for due to the small number of postmenopausal participants and imbalance across groups. Because this cohort was recruited from Israeli centres with a relatively high proportion of OAGB procedures, the external validity of the findings may be limited in settings where SG or RYGB predominate. However, postoperative nutritional guidelines in Israel align closely with internationally accepted protocols (including ASMBS recommendations), and early postoperative physiological responses, such as rapid weight loss, reduced energy and protein intake and early vulnerability of lean mass and bone, are common across bariatric procedures. Therefore, although differences in procedure mix warrant consideration, the mechanistic implications and observed exercise‐related effects are likely relevant across diverse clinical settings.

The study's strengths include its randomized design and detailed body composition and BMD analysis, providing a comprehensive assessment of FFM, FM and BMD changes. We also employed Bayesian analytic techniques that yield actual probabilities of outcomes, avoiding the limitations of null hypothesis testing and reliance on *p*‐values consistent with modern, good statistical practice [27]. Few trials focus on exercise during early post‐MBS weight loss [11], making this study particularly relevant. Moreover, the online intervention reflects real‐world applicability, showing that remote physical activity programmes can be effective for MBS patients, even those with mobility [40], gym access or body image barriers [40].

In summary, combined exercise in the early post‐MBS phase may help optimize body composition, physical function and strength, whereas aerobic training may more effectively attenuate bone loss. These findings suggest that structured, goal‐specific exercise modalities could be beneficial during the early postoperative period to support metabolic, skeletal and functional recovery.

## Funding

This study was financially supported by a grant from the U.S.‐Israel Binational Science Foundation (BSF, Grant No. 2019313), the Israeli Science Foundation (ISF, Grant No. 848/22) and the Recanati Science Foundation (Grant No. 601442001). Additional support for PhD excellence was provided to AG by the Israeli Scholarship Education Foundation (ISEF).

## Ethics Statement

The Ethics Committee of Herzliya Medical Center, Assuta Hospital, and Tel Aviv University approved the study protocol in compliance with the Declaration of Helsinki and its later amendments. All patients gave their informed consent prior to their inclusion in the study.

## Conflicts of Interest

The authors declare no conflicts of interest.

## Supporting information


**Data S1:** Physical activity protocol according to intervention.

**Data S2:** Fat‐free mass loss and physical function according to differences in protein intake.

**Data S3:** Supplementary references.
