# Supplementary material for: Exercise Modalities to Preserve Muscle Mass and Bone Health After Metabolic Bariatric Surgery
Source: J Cachexia Sarcopenia Muscle. 2026 Apr 30;17(3):e70289. doi: 10.1002/jcsm.70289 (PMC13129681; doi:10.1002/jcsm.70289)
Supplement: Supplementary file 1 — Data S1: Physical activity protocol according to intervention. [file JCSM-17-e70289-s002.docx]

**Supplement 1: Physical activity protocol according to intervention**

*Phase 0* **(weeks 3-4 after surgery):** This phase was similar for all intervention groups and consisted of walking for 15-20 minutes, three times per week, at a low intensity of perceived exertion (RPE 2-3 on the Borg scale).

***Aerobic exercise training group.***

Phase 1 **(weeks 5-12 after surgery):** This phase included 30-40 minutes of walking, cycling or other aerobic exercise three times per week at an intensity of 60-70% of peak VO_2_ or RPE 5-7 on the Borg scale.

Phase 2 **(Weeks 13-26 after surgery):** This phase consisted of 60 minutes of exercise, three times per week at an intensity of 70-80% of peak VO_2_ or RPE 7-8 on Borg scale. Exercises included walking, cycling, swimming or other aerobic exercise.

***Resistance exercise training group:***

Phase 1 **(weeks 5-12 after surgery):** This phase consisted of 5 minutes of joint mobility warm-up, followed by 5 multi-joint exercises, with 2-3 sets of 10 to 15 repetitions at RPE 5-7 on the Borg scale.

Phase 2 **(weeks 13-26 after surgery):** This phase included 6 multi-joint exercises, with 3-4 sets of 8 to 12 repetitions at RPE 7-8 on the Borg scale.

***Combined exercise training.***

This program combined aerobic and resistance exercise training sessions.

Phase 1 **(weeks 5-12 after surgery):** Sessions included a 5-minute warm-up, followed by 5 multi-joint exercises for major muscle groups, with 2 sets of 10 to 15 repetitions at RPE 5-7 on the Borg scale. Afterward, the aerobic component of the training included 15-20 minutes of walking, cycling or other aerobic exercise at 60-70% of peak VO_2_ or RPE 5-7 on the Borg scale.

Phase 2 **(weeks 13-26 after surgery):** This phase involved 6 multi-joint exercises with 2-3 sets of 8 to 12 repetitions at an RPE 7-8 on the Borg scale along with 30 minutes of aerobic exercise at 70-80% of peak VO_2_ or RPE 7-8 on the Borg scale.
