# Supplementary material for: Exercise Modalities to Preserve Muscle Mass and Bone Health After Metabolic Bariatric Surgery
Source: J Cachexia Sarcopenia Muscle. 2026 Apr 30;17(3):e70289. doi: 10.1002/jcsm.70289 (PMC13129681; doi:10.1002/jcsm.70289)
Supplement: Supplementary file 2 — Data S2: Fat‐free mass loss and physical function according to differences in protein intake. [file JCSM-17-e70289-s001.docx]

**Supplement 2. Fat-Free Mass Loss and Physical Function According to Differences in Protein Intake**

|  | **Pre-MBS**  **(n=53)**^¥^ | **Postoperative**  **Protein intake**  **<60g/d**  **n=26** | **Postoperative**  **Protein intake >60g/d**  **n=13** | **Postoperative Protein/IBW <1 g/kg IBW/d**  **n=29** | **Postoperative**  **Protein/IBW >1 g/kg IBW/d**  **n=10** |
| --- | --- | --- | --- | --- | --- |
| **Protein (gr.)** | 77.8 ± 32.0 (70.7) | **45.6 ± 11.9 (46.7)^a^** | **77.1 ± 14.8 (73.8)^a^** | **47.8 ± 13.1 (48.5) ^b^** | **80.1 ± 15.6 (76.3) ^b^** |
| **Protein (%)** | 25.5 ± 5.9 (25.5) | **21.7 ± 5.6 (20.9)** | **26.2 ± 4.5 (26.9)** | 22.4 ± 5.7 (21.3) | 25.5 ± 4.9 (25.4) |
| **Δ Hand grip (kg)** | 31.1 ± 11.8 (27.7) | -1.5 ± 5.2 (-1.5) | 1.5 ± 4.6 (2.3) | -1.4 ± 5.0 (-1.5) | 2.0 ± 5.0 (3.3) |
| **Δ RM1/kg body weight leg** | 1.1 ± 0.3 (1.1) | **0.1 ± 0.4 (0.0) ^a^** | **0.4 ± 0.3 (0.3) ^a^** | 0.1 ± 0.4 (0.1) | 0.4 ± 0.3 (0.3) |
| **Δ RM1/kg body weight chest** | 0.3 ± 0.1 (0.3) | **0.0 ± 0.1 (0.0) ^a^** | **0.1 ± 0.1 (0.0) ^a^** | **0.0 ± 0.1 (0.0) ^b^** | **0.1 ± 0.1 (0.1)^b^** |
| **Δ Vo_2_/Kg at 80% Max (ml/Kg/Min)** | 16.1 ± 3.0 (16.0) | 3.5 ± 3.4 (4.3) | 4.5 ± 3.9 (5.1) | 3.5 ± 3.4 (4.3) | 5.0 ± 4.1 (5.2) |
| **Δ Vo_2_/FFM at 80% Max (ml/Kg/Min)** | 32.4 ± 5.8 (31.9) | 0.6 ± 6.4 (1.0) | 2.6 ± 4.7 (4.1) | 0.7 ± 6.2 (1.0) | 2.8 ± 4.8 (4.1) |
| **Δ Sit to stand test (sec.)** | 8.0 ± 2.1 (7.6) | -0.7 ± 2.1 (-0.8) | -1.3 ± 1.3 (-1.7) | -0.7 ± 2.0 (-0.7) | -1.5 ± 1.4 (-2.1) |
| **Δ 6 min. walk test (m)** | 477.7 ± 60.5 (480.2) | 69.4 ± 49.1 (72.0) | 69.4 ± 36.2 (67.5) | 69.3 ± 49.3 (71.1) | 69.9 ± 29.9 (70.5) |

**Abbreviations:** Δ, Change at six months (difference from baseline); FFM, Fat-free mass; IBW, Ideal body weight, calculated as the weight of each individual at a body mass index (BMI) of 25 kg/m²; MBS, Metabolic bariatric surgery; RM1, One-repetition maximum; VO₂, Volume of oxygen consumption. Submaximal VO₂ was assessed at 80% of maximal heart rate predicted during a graded ergometer biking test. Total one-repetition maximum (1RM) is the sum of the maximum weight a participant can lift in one attempt on the chest press and leg press. Differences were compared between two different cutoffs for protein intake: 60 g/day or 1 g of protein per kg of IBW, using the Mann-Whitney test for non-parametric numerical variables. All tests were two-tailed, with statistical significance determined at *p* < 0.05. Significant differences are represented by respective letters and **bold** values. Numerical variables are presented as mean ± standard deviation (median).

^¥^: All variables at baseline are presented as baseline values and not changes.

^a^: Significant differences between those consuming less than 60 gr. of protein per day vs. those consuming >60 gr/day.

^b^: Significant differences between those consuming less than 1 gr/protein per kg. of IBW/ day vs. those consuming >1 gr/ protein per kg. of IBW/day.
