# Supplementary material for: Exercise Modalities to Preserve Muscle Mass and Bone Health After Metabolic Bariatric Surgery
Source: J Cachexia Sarcopenia Muscle. 2026 Apr 30;17(3):e70289. doi: 10.1002/jcsm.70289 (PMC13129681; doi:10.1002/jcsm.70289)
Supplement: Supplementary file 3 — Data S3: Supplementary references. [file JCSM-17-e70289-s003.docx]

**Supplementary references:**

S1 Nuijten MAH, Monpellier VM, Eijsvogels TMH, Janssen IMC, Hazebroek EJ, Hopman MTE. Rate and Determinants of Excessive Fat-Free Mass Loss After Bariatric Surgery. Obes Surg. 2020;30(8):3119-3126. doi:10.1007/s11695-020-04654-6

S2 Marc-Hernandez A, Ruiz-tovar J, Aracil A, Guillén S, Moya-ramón M. Impact of Exercise on Body Composition and Cardiometabolic Risk Factors in Patients Awaiting Bariatric Surgery. Obes Surg. 2019;29(12):3891-3900.

S3 Morales-Marroquin E, Kohl HW, Knell G, de la Cruz-Muñoz N, Messiah SE. Resistance Training in Post-Metabolic and Bariatric Surgery Patients: a Systematic Review. Obes Surg. 2020;30(10):4071-4080. doi:10.1007/s11695-020-04837-1

S4. Shah M, Snell PG, Rao S, et al. High-volume exercise program in obese bariatric surgery patients: A randomized, controlled trial. Obesity. 2011;19(9):1826-1834. doi:10.1038/oby.2011.172

S5 Hangartner TN, Warner S, Braillon P, Jankowski L, Shepherd J. 2013 Position Development Conference on Bone Densitometry The Official Positions of the International Society for Clinical Densitometry : Acquisition of Dual-Energy X-Ray Absorptiometry Body Composition and Considerations Regarding Analysis and Repeatabil. J Clin Densitom. 2024;16(4):520-536. doi:10.1016/j.jocd.2013.08.007

S6 Rehm CD, Peñalvo JL, Afshin A, Mozaffarian D. Dietary intake among US Adults, 1999-2012. JAMA - J Am Med Assoc. 2016;315(23):2542-2553. doi:10.1001/jama.2016.7491

S7 Goshen A, Goldbourt U, Shohat T, Shimony T, Keinan-Boker L, Gerber Y. Diet quality in relation to healthy ageing: the Israeli Longitudinal Study on Aging (ILSA)-a study protocol. BMJ Open. 2019;9(4). doi:10.1136/BMJOPEN-2018-024673

S8 Van Buuren S, Groothuis-Oudshoorn K. mice: Multivariate imputation by chained equations in R. *J Stat Softw*. 2011;45(3):1-67. doi:10.18637/jss.v045.i03

S9 Greenland S, Senn SJ, Rothman KJ, et al. Statistical tests, P values, confidence intervals, and power: a guide to misinterpretations. *Eur J Epidemiol*. 2016;31(4):337-350. doi:10.1007/s10654-016-0149-3

S10 Kruschke JK, Liddell TM. The Bayesian New Statistics: Hypothesis testing, estimation, meta-analysis, and power analysis from a Bayesian perspective. *Psychon Bull Rev*. 2018;25(1):178-206. doi:10.3758/s13423-016-1221-4

S11 Spiegelhalter DJ, Abrams KR, Myles JP. Bayesian Approaches to Clinical Trials and Health-Care Evaluation. *Bayesian Approaches to Clin Trials Heal Eval*. Published online 2004:1-392. doi:10.1002/0470092602

S12 Bittl JA, He Y. Bayesian Analysis: A Practical Approach to Interpret Clinical Trials and Create Clinical Practice Guidelines. *Circ Cardiovasc Qual Outcomes*. 2017;10(8):1-11. doi:10.1161/CIRCOUTCOMES.117.003563

S13 Bürkner PC. brms: An R package for Bayesian multilevel models using Stan. *J Stat Softw*. 2017;80(1). doi:10.18637/jss.v080.i01

S14 Bürkner PC. Advanced Bayesian multilevel modeling with the R package brms. *R J*. 2018;10(1):395-411. doi:10.32614/rj-2018-017

S15 Murai IH, Roschel H, Dantas WS, et al. Exercise Mitigates Bone Loss in Women with Severe Obesity after Roux-en-Y Gastric Bypass: A Randomized Controlled Trial. J Clin Endocrinol Metab. 2019;104(10):4639-4650. doi:10.1210/jc.2019-00074

S16 Sahni S, Cupples LA, McLean RR, et al. Protective effect of high protein and calcium intake on the risk of hip fracture in the Framingham Offspring cohort. J Bone Miner Res. 2010;25(12):2770-2776. doi:10.1002/jbmr.194

S17 Bond DS, Wu Y, Baillot A, Lillis J, Sundgot-Borgen C, Papasavas PK. The Role of Physical Activity in Minimizing Recurrence of Weight Gain Following Metabolic and Bariatric Surgery: Current Evidence and Suggestions for Advancing Future Research. Curr Obes Rep. 2025 Aug 21;14(1):66. doi: 10.1007/s13679-025-00658-4. PMID: 40836203; PMCID: PMC12380178.
